# A STATEWIDE PROGRAM FOR SCREENING FOR DEMENTIA IN MEDI-CAL PRIMARY CARE IN CALIFORNIA: DEMENTIA CARE AWARE

**DOI:** 10.1093/geroni/igae098.0422

**Published:** 2024-12-31

**Authors:** Anna Chodos

**Affiliations:** University of California San Francisco School of Medicine, San Francisco, California, United States

## Abstract

In California, the Department of Health Care Services (Medi-Cal) expanded benefits for older beneficiaries to include screening for dementia by trained primary providers starting in July 2022. In addition, they created a program to promote this screening and provide the training called Dementia Care Aware. The University of California, San Francisco was awarded the contract to realize this program through the end of September 2024 and brought together 4 other University of California campuses and statewide Alzheimer’s organizations to stand up the program. During the symposium, attendees will hear about the key accomplishments from creating and disseminating this program. These include: the development of the screening approach, called the cognitive health assessment, with state and national experts; the creation of interactive on-line learning and live trainings; success in dissemination to California’s 58 counties and overall uptake among providers and progress to the program’s goals of 4000 trained in two years; and lessons learned. Implementation of dementia screening based on the cognitive health assessment in two public health systems (in San Francisco and Los Angeles) provide evidence of how screening is possible in safety net primary care. Finally, a summary of key challenges will show how common barriers in primary care nationwide can be considered before implementing dementia screening programs.

